# Assessing population genetic structure via the maximisation of genetic distance

**DOI:** 10.1186/1297-9686-41-49

**Published:** 2009-11-09

**Authors:** Silvia T Rodríguez-Ramilo, Miguel A Toro, Jesús Fernández

**Affiliations:** 1 Departamento de Mejora Genética Animal. Instituto Nacional de Investigación y Tecnología Agraria y Alimentaria (INIA). Crta. A Coruña Km. 7,5. 28040 Madrid, Spain; 2 Departamento de Bioquímica, Genética e Inmunología, Facultad de Biología, Universidad de Vigo, 36310 Vigo, Spain; 3 Departamento de Producción Animal, ETS Ingenieros Agrónomos, Universidad Politécnica de Madrid, Ciudad Universitaria, 28040 Madrid, Spain

## Abstract

**Background:**

The inference of the hidden structure of a population is an essential issue in population genetics. Recently, several methods have been proposed to infer population structure in population genetics.

**Methods:**

In this study, a new method to infer the number of clusters and to assign individuals to the inferred populations is proposed. This approach does not make any assumption on Hardy-Weinberg and linkage equilibrium. The implemented criterion is the maximisation (via a *simulated annealing *algorithm) of the averaged genetic distance between a predefined number of clusters. The performance of this method is compared with two Bayesian approaches: STRUCTURE and BAPS, using simulated data and also a real human data set.

**Results:**

The simulations show that with a reduced number of markers, BAPS overestimates the number of clusters and presents a reduced proportion of correct groupings. The accuracy of the new method is approximately the same as for STRUCTURE. Also, in Hardy-Weinberg and linkage disequilibrium cases, BAPS performs incorrectly. In these situations, STRUCTURE and the new method show an equivalent behaviour with respect to the number of inferred clusters, although the proportion of correct groupings is slightly better with the new method. Re-establishing equilibrium with the randomisation procedures improves the precision of the Bayesian approaches. All methods have a good precision for *F*_*ST *_≥ 0.03, but only STRUCTURE estimates the correct number of clusters for *F*_*ST *_as low as 0.01. In situations with a high number of clusters or a more complex population structure, MGD performs better than STRUCTURE and BAPS. The results for a human data set analysed with the new method are congruent with the geographical regions previously found.

**Conclusion:**

This new method used to infer the hidden structure in a population, based on the maximisation of the genetic distance and not taking into consideration any assumption about Hardy-Weinberg and linkage equilibrium, performs well under different simulated scenarios and with real data. Therefore, it could be a useful tool to determine genetically homogeneous groups, especially in those situations where the number of clusters is high, with complex population structure and where Hardy-Weinberg and/or linkage equilibrium are present.

## Background

Traditional population genetic analyses deal with the distribution of allele frequencies between and within populations. From these frequencies several measures of population structure can be estimated, the most widely used being the Wright *F *statistics [1]. To calculate these estimators of population structure an *a priori *definition of the population is needed. Population determination is usually based on phenotypes or the geographical origin of samples. However, the genetic structure of a population is not always reflected in the geographical proximity of individuals. Nevertheless, populations that are not discretely distributed can be genetically structured, due to unidentified barriers to gene flow. In addition, in groups of individuals with different geographical locations, behavioural patterns or phenotypes are not necessarily genetically differentiated [2]. As a consequence, an inappropriate *a priori *grouping of individuals into populations may diminish the power of the analyses to elucidate biological processes, potentially leading to unsuitable conservation or management strategies.

Bayesian clustering algorithms [3-6] have recently emerged as a prominent computational tool to infer population structure in population genetics and in molecular ecology [7]. These methods use genetic information to ascertain population membership of individuals without assuming predefined populations. They can assign either the individuals or a fraction of their genome to a number of clusters (*K*) based on multilocus genotypes. The methods operate by minimising Hardy-Weinberg and linkage disequilibrium (but the assumption of Hardy-Weinberg equilibrium within clusters could be avoided, see [8]). The procedures generally involve Markov chain Monte Carlo (MCMC) approaches. These particular clustering methods are useful when genetic data for potential source populations are not available (in opposition to assignment methods), and they offer a powerful tool to answer questions of ecological, evolutionary, or conservation relevance [9].

A recent study by Latch *et al*. [10] compared the relative performance of three non-spatial Bayesian clustering programs, STRUCTURE [3], PARTITION [4] and BAPS [5]. A significant difference between STRUCTURE and PARTITION programs is that the former allows the presence of admixed individuals while the latter assumes that all individuals are of pure ancestry. Two main features distinguish BAPS from STRUCTURE. First, in BAPS the number of populations is treated as an unknown parameter that could be estimated from the data set. Second, in the BAPS version 2 a stochastic optimisation algorithm is implemented to infer the posterior mode of *K *instead of the MCMC algorithm also used in STRUCTURE. Notwithstanding, the most widely used genotypic clustering method is that implemented in the program STRUCTURE.

Other clustering methods implement a maximum likelihood method using an expectation-maximisation algorithm, to infer population stratification and individual admixture [11,12].

Current developments of Bayesian clustering methods explicitly address the spatial nature of the problem of locating genetic discontinuities by including the geographical coordinates of individuals in their prior distributions [13-15]. Another way to proceed, as a complement to the previous approaches, is to look directly for the zones of sharp change in genetic data. Two approaches seem better adapted to analyse genetic data: the Wombling method [16] and the Monmonier algorithm [17-19].

Another approach, proposed by Dupanloup *et al. *[17], is a spatial procedure (spatial analysis of molecular variance; SAMOVA) that does not make any assumption on Hardy-Weinberg equilibrium (HWE) and linkage equilibrium (LE). SAMOVA uses a *simulated annealing *algorithm to find the configuration that maximises the proportion of total genetic variance due to differences between groups of populations (a higher hierarchical level when comparing to the alternative group of individuals). In the starting steps of the SAMOVA method, a set of Voronoi polygons are constructed from the geographical coordinates of the sampled points. Thus, this procedure can be useful to identify the location of barriers to gene flow between groups.

In the present study, a simple and general method to infer the population structure by assigning individuals to the inferred subpopulations is proposed. The new approach, that implements a *simulated annealing *algorithm, is based on the maximisation of the averaged genetic distance between populations and does not make any assumption on HWE within populations and LE between loci. The performance of this method is compared with two Bayesian clustering methods. Simulated data were used to mimic different scenarios including SNP or microsatellite data. In addition, the performance of the proposed method was tested in a previously analysed human data set.

## Methods

### Bayesian clustering methods

The programs used were STRUCTURE version 2.1 [3,20] and BAPS version 4.14 [5,21,22]. The software PARTITION [4] was not applied in this study because Latch *et al*. [10] have shown that its performance is less good (*e.g. *this method identifies correctly only the number of subpopulations at levels *F*_*ST *_≥ 0.09, while, STRUCTURE and BAPS determine the population substructure extremely well at *F*_*ST *_= 0.02 - 0.03).

The parameters for the implementation of STRUCTURE comprise a burn-in of 10000 replicates following 50000 replicates of MCMC. Specifically, the admixture model and the option of correlated allele frequencies between populations were selected, since this configuration is considered the best by Falush *et al. *[20] in cases of subtle population structures. Similarly, the degree of admixture (alpha) was inferred from the data. When alpha is close to zero, most individuals are essentially from one population or another, while alpha > 1 means that most individuals are admixed. Lambda, the parameter of the Dirichlet distribution of allelic frequencies, was set to one, as advised by the STRUCTURE manual. For each data set, five runs were carried out for each possible number of clusters (*K*) in order to quantify the variation in the likelihood of the data for a given *K*. The range of tested *K *was set according to the true number of simulated populations (see below the simulated data section). Each data set took between 5 to 30 hours to run depending on the number of markers and individuals simulated in the data set (all times provided correspond to a computer with a 3 GHz processor and 2 GB of RAM).

The criterion implemented in STRUCTURE to determine *K *is the likelihood of the data for a given *K*, *L*(*K*). The number of subpopulations is identified using the maximal value of this likelihood returned by STRUCTURE. However, it has been observed that once the real *K *is reached the likelihood at larger *K *levels off or continues increasing slightly, and the variance between runs increases [23]. Consequently, in our work, the distribution of *L*(*K*) did not show a clear mode for the true *K*. Notwithstanding, an *ad hoc *quantity based on the second order rate of change of the likelihood function with respect to *K *(Δ*K*) did show a clear peak at the true value of *K*. Evanno *et al. *[23] have suggested to estimate Δ*K *as

where *avg *is the arithmetic mean across replicates and *sd *is the standard deviation of the replicated *L*(*K*). The value of *K *selected will correspond to the modal value of the distribution of Δ*K*. The grouping analysis was performed on the results from the run with the maximal value of the likelihood of the data for the estimated *K*.

BAPS software was run setting the maximum number of clusters to 20 or 30 depending on the scenario. To make the results fully comparable with those from STRUCTURE, the clustering of the individual option was applied for every scenario. Each data set required approximately 1 to 5 minutes to complete.

### Maximisation of the genetic distance method

The rationale behind the new approach (MGD thereafter) is that highly differentiated populations are expected to show a high genetic distance between them. This distance can be calculated from the molecular marker information without assumptions on HWE or LE.

From all the genetic distances previously published in the literature [24], one of the most used is the Nei minimum distance [25]. One of the advantages of this genetic distance is that it can be calculated through the pairwise coancestry between individuals [26]. Following Nei, the distance between clusters *A *and *B *can be calculated as

where

with *L *the number of loci, *a *the number of alleles in each locus and *p*_*Ajk *_the frequency of allele *k *in the locus *j *for group *A*. The average distance over the entire metapopulation is

where the summation is for all couples of *n *subpopulations, *N*_*i *_is the number of individuals of population *i*, and .

An alternative way of calculating the genetic distance is through the pairwise coancestry between individuals [26]. In this approach, the Nei minimum distance between two subpopulations can be expressed as

where *f*_*AA *_is the average molecular coancestry between individuals of subpopulation *A *and *f*_*AB *_is the average pairwise molecular coancestry between all possible couples of individuals, one from subpopulation *A *and the other from subpopulation *B*.

The molecular coancestry (*f*) can be computed applying Malécot's [27] definition of genealogical coancestry to the molecular marker loci (microsatellites or SNP). Thus, the molecular coancestry at a particular locus between two individuals is calculated as the probability that two alleles taken at random, one from each individual, are equal (identical by state, *IBS*). Throughout several markers, the molecular coancestry is obtained as the arithmetic mean over marker loci.

The advantage of this approach is that the molecular coancestry matrix has to be calculated only once (at the beginning of the optimisation) and then the value for different configurations can be calculated just by averaging different groups of couples. This makes the process quite efficient in terms of computation speed. Notwithstanding, a shortcoming of the method is that no measure of confidence is obtained for the final arrangement of clusters.

This problem can be circumvented when using the allele frequency approach by implementing the following strategy. The considered configurations, instead of assigning each individual to a single cluster, are lists of vectors (one for each individual) carrying their probability to belong to each cluster. Consequently, the sum of positions (*i.e. *probabilities) for a particular individual equals one. In the final (optimal) configuration those individuals with a probability close to one of belonging to a particular cluster can be assigned with great confidence. Contrarily, assignment of individuals with lower probabilities will not be clear, possibly reflecting the presence of admixture or the insufficient amount of information to assign this individual to a single cluster.

To determine the frequency of each allele within a cluster, in order to calculate the genetic distances, the number of copies of that allele carried by each individual has to be multiplied by the probability of the individual belonging to the cluster and summed up across all the individuals in the same cluster. After this has been done with all the alleles in a locus, frequencies must be standardised to guaranty that the sum of allelic frequencies equals one. The disadvantage of this strategy is that it is computationally very demanding, since frequencies have to be recalculated for all the loci and alleles for each new considered configuration. Therefore, calculations take much more time depending on how large is the number of loci and their degree of polymorphism.

### Optimisation procedure

The implementation of both MGD approaches used a *simulated annealing *algorithm to find the partition that showed the maximal average genetic distance between populations. *Simulated annealing *is an optimisation technique initially proposed by Metropolis *et al. *[28]. The connection between this algorithm and mathematical optimisation procedures was noted by Kirkpatrick *et al. *[29]. A more detailed explanation of the application of *simulated annealing *to other genetic issues can be found, for example, in Fernández and Toro [30].

The implementation of the MGD method was done using a tailored program in FORTRAN. The *simulated annealing *algorithm starts from an initial solution obtained by randomly separating individuals into *K *groups (*i.e. K *is predefined in each run of the algorithm) or assigning to each individual a random probability of belonging to each group, if the allele frequency option is selected. Alternative solutions consist in moving one of the individuals from its present cluster to a randomly selected group (when dealing with the molecular coancestry matrix) or in increasing by 0.1% the probability of belonging to one group and decreasing by 0.1% the probability for the same individual of belonging to another cluster. A restriction was included imposing that all groups include at least a representation from one individual.

The values of the actual and the alternative solutions (*i.e. *the averaged genetic distance calculated from whatever strategy considered) were calculated. Due to its nature, *simulated annealing *is a minimisation algorithm but the genetic distance is a parameter to be maximised. Therefore, the sign of both distances must be changed in order to find the desired optimum. Acceptance of the alternative solution occurred with a probability calculated as

where *I *was the difference between values of the alternative and actual solutions and *T *was the present temperature in the particular cooling cycles.

Fifty thousand alternative solutions were generated and tested. Afterwards, the value of *T *was reduced by a factor of *Z*. Another 50000 solutions were generated, the parameter *T *was reduced and so on. A maximum of 400 steps (*i.e. *different values for *T*) were allowed. The rate of decrease in the cooling factor or temperature (*Z*) and the initial temperature were set to 0.9 and 0.001, respectively, based on previous simulations performed to adjust the algorithm in this specific kind of data set. For each scenario, different *K *were tested, and for each *K*, five replicates (starting from different initial solutions) were carried out, as a security measure, in order to avoid being stuck in non-optimal solutions; the replicate with the highest genetic distance was chosen for the grouping analysis. Each run of the program took between 1 to 8 hours to complete when the genetic distance was calculated from the molecular coancestry. However, if the genetic distance was calculated from the allele frequencies the computation time suffered a10-fold increase. In this paper, only the results obtained with the allele frequency strategy are presented, because both approaches showed similar accuracies in the tested situations.

**Figure 1:**
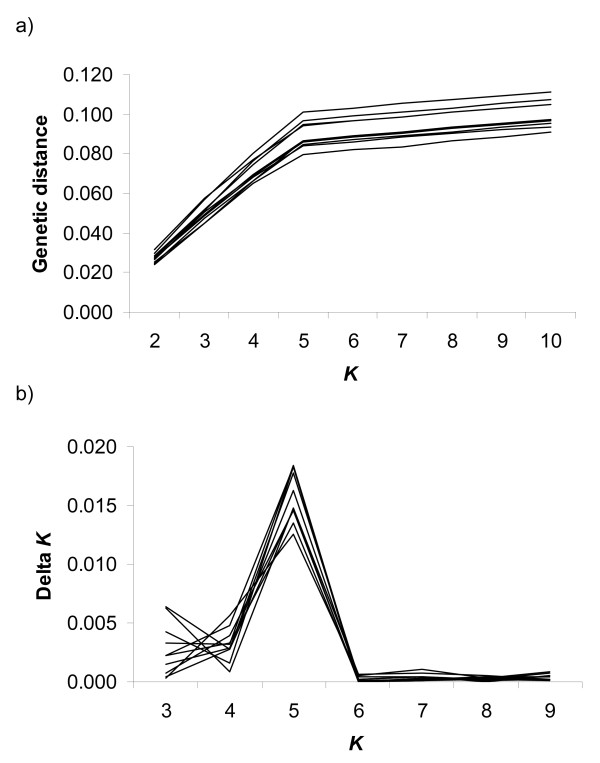
**Genetic distance (a) and Δ*K *(b) against the cluster number**. Example of ten replicates of a single scenario (*K *= 5).

As for the likelihood in STRUCTURE, the values for the averaged genetic distance did not reach a clear maximum in a sensible range of successive *K *values (*i. e. *continued increasing slightly after the true number of clusters had been reached). For this reason, a similar procedure as that proposed in Evanno *et al. *[23] for STRUCTURE was implemented. It was based on the rate of change in the averaged genetic distance between successive *K *values (Δ*K*) calculated as

where *D *is the averaged genetic distance in the optimal solution for a given *K*. The inferred number of clusters corresponds to the value with the highest Δ*K*. Figure 1 shows values of genetic distance for the different *K *and the corresponding transformed values Δ*K *used to determine the correct grouping (values for 10 replicates of the same scenario).

Another appealing objective of this study would have been to compare the results obtained with MGD and SAMOVA software since both are methods free of assumptions about the equilibriums and use a similar approach to perform the clusterisation. However, such an evaluation is not possible due because SAMOVA is a method that clusters populations whereas the MGD method clusters individuals, which makes any comparison between the two approaches difficult.

### Simulated data

To generate genotypic data, the EASYPOP software version 1.7 [31] was used. The modelled organisms were diploid, hermaphroditic and randomly mated (excluding selfing, except when indicated). The population comprised five subpopulations with an equal number of individuals constant along the generations. A finite island model of migration was simulated, where each of the subpopulations exchanged migrants at a rate *m *= 0.01 per generation to a random chosen subpopulation.

The simulated mutational model assumed equal probability of mutating to any allelic state (*KAM*). Alleles at the base population were randomly assigned, and thus, frequencies of all alleles were initially equal. Free recombination was considered between loci. The evaluated populations covered a broad range of scenarios with various degrees of differentiation and depending on whether they were in mutation-migration-drift equilibrium or not. The parameter set for the simulations are summarised in Table 1. The parameters involved were the following:

**Table 1:** Parameter set, genetic variability values and Wright *F *statistics considered in each evaluated scenario

	Microsatellite loci			
	
Scenario	1	2	3	4
Generations	10000	10000	20	20
Subpopulation size	100	100	20	20
Number of markers	10	50	10	50
Number of alleles	10	10	10	10
				
Genetic variability:				
*n*_*a*_	7.72 ± 0.14	7.78 ± 0.05	8.79 ± 0.11	8.66 ± 0.05
*H*_*O*_	0.55 ± 0.02	0.56 ± 0.01	0.59 ± 0.01	0.60 ± 0.01
*H*_*S*_	0.56 ± 0.02	0.56 ± 0.01	0.59 ± 0.01	0.60 ± 0.01
*H*_*T*_	0.64 ± 0.02	0.65 ± 0.01	0.82 ± 0.00	0.83 ± 0.00
				
Wright *F *statistics:				
*F*_*IS*_	0.01 ± 0.00	0.00 ± 0.00	0.01 ± 0.01	0.00 ± 0.01
*F*_*ST*_	0.13 ± 0.00	0.13 ± 0.00	0.27 ± 0.01	0.27 ± 0.01
*F*_*IT*_	0.13 ± 0.01	0.12 ± 0.01	0.28 ± 0.01	0.28 ± 0.01

	**SNP loci**			
	
**Scenario**	**5**	**6**	**7**	**8**

Generations	1000	1000	20	20
Subpopulation size	100	100	20	20
Number of markers	60	300	60	300
Number of alleles	2	2	2	2
				
Genetic variability:				
*n*_*a*_	1.53 ± 0.10	1.60 ± 0.01	2.00 ± 0.00	2.00 ± 0.00
*H*_*O*_	0.19 ± 0.01	0.18 ± 0.00	0.33 ± 0.01	0.33 ± 0.00
*H*_*S*_	0.19 ± 0.01	0.18 ± 0.00	0.33 ± 0.00	0.33 ± 0.00
*H*_*T*_	0.22 ± 0.01	0.21 ± 0.00	0.46 ± 0.00	0.46 ± 0.00
				
Wright *F *statistics:				
*F*_*IS*_	0.00 ± 0.00	0.00 ± 0.00	0.01 ± 0.01	0.00 ± 0.00
*F*_*ST*_	0.14 ± 0.01	0.14 ± 0.00	0.27 ± 0.01	0.27 ± 0.01
*F*_*IT*_	0.14 ± 0.01	0.14 ± 0.00	0.28 ± 0.01	0.27 ± 0.01

The following parameters were fixed in all data sets: diploidy, hermaphroditic, random mating, finite island model, five subpopulations, equal number of individuals in all subpopulations, constant population size, migration rate *m *= 0.01, *KAM *mutation model, equal frequencies for all allelic states in the initial population, free recombination between loci, mutation rate: 10^-3 ^for microsatellite loci and 5 × 10^-7 ^for SNP loci. *n*_*a*_: number of alleles; *H*_*O*_: observed heterozygosity; *H*_*S*_: mean subpopulation gene diversity; *H*_*T*_: mean total gene diversity

1. Individuals in each subpopulation: 20 or 100.

2. Allelic states: 10 for the microsatellite-like markers and two for the SNP.

3. Available molecular markers: 10 or 50 for the microsatellites and 60 or 300 for the SNP.

4. Mutation rate: 10^-3 ^for the microsatellite and 5 × 10^-7 ^for the SNP.

5. Number of generations elapsed since foundation: 20, 1000 or 10000.

Table 1 also shows the values for some diversity and Wright *F *statistics in each evaluated scenario.

In addition, to test in depth the efficiency of the methods, some simulations were performed with modified scenarios involving several factors like the level of differentiation, the size or complexity of the metapopulation and the presence of Hardy-Weinberg and/or linkage disequilibrium (HWD and LD). The additional situations were the following:

1. Scenario 2 with *m *= 0.05, *m *= 0.07 and *m *= 0.10 to evaluate different *F*_*ST *_values.

2. Scenario 2 with 10 subpopulations (*K *= 10) and with 50 individuals in each subpopulation to test the efficiency of the algorithms when the number of clusters is large. In this scenario, *K *values ranging from 5 to 15 were tested.

3. Hierarchical island model (HIM) consists in five sets of four subpopulations, each made of 50 individuals. Migration occurs at a rate of 0.02 within a given archipelago and 0.001 between archipelagos. Fifty microsatellites and 300 SNP were tested for *K *values ranging from 2 to 23 both for STRUCTURE and MGD, and BAPS software was run setting the maximum number of clusters to 30 because in this scenario the total number of subpopulations could reach 20 (not just 5).

4. Scenario 3 with a proportion of selfing equal to 0.3, 0.5, 0.7 and 0.9 to generate Hardy-Weinberg disequilibrium.

5. Scenario 6 considering 1000 generations where migration was not allowed followed by 10 generations where *m *= 0.01 or *m *= 0.1. To generate linkage disequilibrium during the 1010 generations, the recombination rate between loci was set to 0.06. This value of recombination rate was calculated according to the Haldane mapping function [32] considering a very small genome (around 20 centimorgans) in order to generate a tight linkage between each marker (300 SNP).

Parameters corresponding to the above situations are given in Table 2. Ten replicated data sets were tested for all scenarios.

**Table 2:** Genetic variability and Wright statistics with different migrations, *K *= 10, HIM, HWD and LD

	Scenario 2				HIM	
		
	*m *= 0.05	*m *= 0.07	*m *= 0.10	*K *= 10	50 markers	300 markers
Genetic variability:						
*n*_*a*_	7.68 ± 0.04	7.75 ± 0.08	7.73 ± 0.05	8.03 ± 0.04	9.58 ± 0.03	1.14 ± 0.02
*H*_*O*_	0.60 ± 0.01	0.61 ± 0.01	0.62 ± 0.01	0.49 ± 0.00	0.50 ± 0.00	0.02 ± 0.00
*H*_*S*_	0.60 ± 0.01	0.62 ± 0.01	0.63 ± 0.01	0.50 ± 0.00	0.51 ± 0.00	0.02 ± 0.00
*H*_*T*_	0.62 ± 0.01	0.63 ± 0.01	0.63 ± 0.01	0.67 ± 0.00	0.79 ± 0.00	0.05 ± 0.00
						
Wright *F *statistics:						
*F*_*IS*_	0.00 ± 0.00	0.00 ± 0.00	0.01 ± 0.00	0.01 ± 0.00	0.01 ± 0.00	0.01 ± 0.00
*F*_*ST*_	0.03 ± 0.00	0.02 ± 0.00	0.01 ± 0.00	0.26 ± 0.01	0.35 ± 0.00	0.50 ± 0.01
*F*_*IT*_	0.03 ± 0.00	0.02 ± 0.00	0.02 ± 0.00	0.27 ± 0.01	0.36 ± 0.00	0.50 ± 0.01

	**Scenario 3 (HWD)**				**Scenario 6 (LD)**	
		
	***s *= 0.3**	***s *= 0.5**	***s *= 0.7**	***s *= 0.9**	***m *= 0.01**	***m *= 0.1**

Genetic variability:						
*n*_*a*_	8.45 ± 0.17	8.21 ± 0.08	7.78 ± 0.19	7.18 ± 0.19	1.95 ± 0.00	1.94 ± 0.00
*H*_*O*_	0.51 ± 0.01	0.39 ± 0.01	0.23 ± 0.01	0.09 ± 0.01	0.08 ± 0.01	0.36 ± 0.00
*H*_*S*_	0.60 ± 0.01	0.54 ± 0.01	0.48 ± 0.02	0.46 ± 0.02	0.09 ± 0.01	0.37 ± 0.00
*H*_*T*_	0.82 ± 0.00	0.81 ± 0.00	0.81 ± 0.01	0.79 ± 0.01	0.40 ± 0.00	0.40 ± 0.00
						
Wright *F *statistics:						
*F*_*IS*_	0.15 ± 0.01	0.29 ± 0.01	0.52 ± 0.01	0.81 ± 0.02	0.12 ± 0.02	0.02 ± 0.00
*F*_*ST*_	0.27 ± 0.01	0.33 ± 0.01	0.40 ± 0.02	0.42 ± 0.02	0.76 ± 0.02	0.07 ± 0.00
*F*_*IT*_	0.38 ± 0.01	0.52 ± 0.01	0.71 ± 0.01	0.89 ± 0.01	0.79 ± 0.02	0.09 ± 0.01

Scenario 2 simulated with different migration rates (*m*) and a higher number of subpopulations (*K *= 10); hierarchical island model (HIM) with 50 microsatellites and 300 SNP; scenario 3 simulated with selfing (0.3, 0.5, 0.7 and 0.9) to generate Hardy-Weinberg disequilibrium (HWD); scenario 6 with linked loci (recombination rate = 0.06) and 1000 generations with no migration between subpopulations and 10 generations where *m *= 0.01 or *m *= 0.1 to generate linkage disequilibrium (LD); see Table 1 for abbreviations and for the explanation of scenarios

GENEPOP software version 4.0.6 [33] was used to analyse Hardy-Weinberg and/or linkage equilibrium (or disequilibrium) in scenarios 3 and 6. To compute HWE, the option *F*_*ST*_*and other correlations, isolation by distance *was chosen with the suboption of *all populations*. The Wright *F *statistic [1]*F*_*IS *_is provided. Regarding the LE, the option of the *exact test for genotypic disequilibrium *was selected with the suboption of *test for each pair of loci in each subpopulation*. A *P*-value for each pair of loci is computed for all subpopulations (Fisher method), and the high (or reduced) proportion of significant loci pairs (*P *< 0.05) with significant linkage is a measure of the LD (or LE). The data sets corresponding to scenarios 3 and 6 in Table 1 show no significant departures from Hardy-Weinberg and linkage equilibrium (*F*_*IS *_= 0.01 ± 0.01 and 0.00 ± 0.00 for scenarios 3 and 6, respectively). The mean proportions of significant loci pairs with significant linkage are 0.12 ± 0.01 and 0.07 ± 0.00 for scenarios 3 and 6, respectively. The data sets corresponding to modified scenarios 3 and 6 in Table 2 show both significant departures from Hardy-Weinberg and linkage equilibrium. The mean *F*_*IS *_values range from 0.15 ± 0.01 to 0.81 ± 0.02 in scenario 3. The mean proportions of significantly linked loci pairs are 0.35 ± 0.05, 0.60 ± 0.08, 0.88 ± 0.02 and 0.99 ± 0.00 with a proportion of selfing equal to 0.3, 0.5, 0.7 and 0.9, respectively. The mean *F*_*IS *_values are 0.12 ± 0.02 and 0.02 ± 0.00 in scenario 6 with *m *= 0.01 and *m *= 0.1, respectively. The mean proportions of significantly linked loci pairs are 0.73 ± 0.01 and 0.22 ± 0.01 in scenario 6 with *m *= 0.01 and *m *= 0.1, respectively.

### Randomisation procedure

As an example, to determine the relative influence of HWD and LD in the accuracy of the evaluated methods, the data of those replicates where both STRUCTURE and BAPS failed to estimate the correct number of clusters in scenario 3 with *s *= 0.7 and scenario 6 with *m *= 0.01 were randomised to re-establish HWE and/or LE. This procedure was implemented since HWD and LD could interfere in the performance of the Bayesian approaches. The expectation was that after the randomisation procedures the Bayesian approaches could perform better because HWE and LE are assumptions for both methodologies.

Three alternatives were followed to randomise the data within subpopulations. First, an allele randomisation to re-establish HWE and LE in the data sets. Second, between loci genotypes were also randomised to maintain HWD while restoring LE. Finally, haplotypes were also taken haphazardly to evaluate the opposite situation (HWE and LD). GENEPOP confirmed Hardy-Weinberg and linkage equilibrium (or disequilibrium) after the randomisation of alleles, genotypes or haplotypes.

### Measures of accuracy

To determine the performance of each method the number of inferred clusters (*K*) was evaluated through the modal value over replicates and, also, with the fraction of replicates where the estimated number of clusters was inferred to be the true number. A more detailed measure can be obtained as the proportion of individuals correctly grouped with their true population. This parameter was evaluated by averaging over clusters the highest proportion of each subpopulation (*i.e. *larger group of individuals) located at the same cluster. This mean value was also averaged over replicates.

### Real data

The MGD method was also tested on a real data set of 1056 humans subdivided into 52 populations genotyped for 377 microsatellite loci obtained from http://rosenberglab.bioinformatics.med.umich.edu/diversity.html#data1. This data set was previously examined both with STRUCTURE [34] and BAPS [21]. Since Rosenberg *et al*. [34] ran STRUCTURE up to *K *= 6 we re-ran STRUCTURE for *K *= 7 with the parameters proposed by Rosenberg *et al*. [34] to compare the results obtained from the three methodologies.

## Results

The performances under the allelic frequency approach and the molecular coancestry approach where similar and, thus, only the former will be shown.

### Simulated data

The number of inferred clusters in each simulated scenario for the evaluated methods is given in Table 3. When the modal value was the comparison criterion, both STRUCTURE and MGD had an optimal behaviour in the simulated scenarios since they always yielded the true number of subpopulations. BAPS overestimated the number of populations when a reduced number of molecular information was available. When the fraction of replicates with the correct number of clusters estimated was the comparison parameter, MGD performed slightly better than BAPS and STRUCTURE. Generally, all methods increased their accuracy when a large number of markers were available and after a huge number of generations (*i.e. *when mutation-migration-drift was reached).

**Table 3:** Modal value and fraction of replicates where the estimated number of clusters (*K*) was 5

	Microsatellite loci				SNP loci			
		
Scenario	1	2	3	4	5	6	7	8
Modal value:								
*STRUCTURE*	5	5	5	5	5	5	5	5
*BAPS*	10	5	6	5	14	5	6	5
*MGD*	5	5	5	5	5	5	5	5
								
Replicates *K *= 5:								
*STRUCTURE*	0.7	1.0	0.9	0.4	0.6	1.0	0.8	0.8
*BAPS*	0.0	1.0	0.3	0.6	0.0	0.9	0.0	0.4
*MGD*	0.8	1.0	1.0	1.0	0.9	1.0	1.0	1.0

See Table 1 for the explanation of scenarios

**Figure 2:**
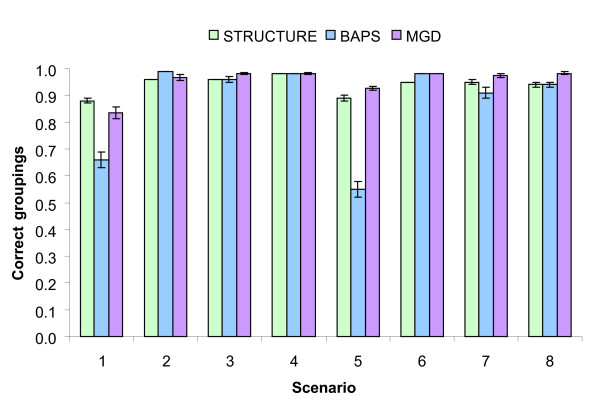
**Mean proportion of correct groupings over replicates in each scenario and method**. Bars represent standard errors; see Table 1 for the explanation of the scenarios.

Figure 2 shows the averaged proportion of correct groupings over replicates. With all the methods more than 80% of the individuals were assigned to the correct cluster. However, a smaller percentage was observed with BAPS in situations with a reduced number of markers even if a large number of generations elapsed. In general, the MGD method performed slightly better, although there were no significant differences between the approaches across scenarios.

The influence of the different factors underlined above in the inference of the substructure is shown in Table 4. When modal values were compared, STRUCTURE performed better regarding the differentiation level (it always predicted the correct number of clusters), whereas BAPS and MGD were equivalent and underestimated *K *when *m *= 0.10. Contrarily, when *K *= 10, BAPS and MGD performed better than STRUCTURE. In HIM, both STRUCTURE and MGD indicate five clusters and BAPS gives an overestimation. It should be pointed out that, although the highest Δ*K *in this scenario was obtained for *K *= 5 under MGD, a smaller 'peak' was observed for *K *= 20, and thus it also detected the structure at the lower level (data not shown).

**Table 4:** Modal value and fraction of replicates where *K *= 5 (10) in the remaining scenarios

	Scenario 2				HIM	
		
	*m *= 0.05	*m *= 0.07	*m *= 0.10	*K *= 10	50 markers	300 markers
Modal value:						
*STRUCTURE*	5	5	5	9	5	5
*BAPS*	5	5	3	10	21	18
*MGD*	5	5	3	10	5	5
						
Replicates *K *= 5 (or 10):						
*STRUCTURE*	1.0	1.0	0.9	(0.2)	0.6	0.7
*BAPS*	1.0	0.9	0.3	(0.6)	0.0	0.0
*MGD*	0.9	0.5	0.2	(1.0)	1.0	1.0

	**Scenario 3 (HWD)**				**Scenario 6 (LD)**	
		
	***s *= 0.3**	***s *= 0.5**	***s *= 0.7**	***s *= 0.9**	***m *= 0.01**	***m *= 0.1**

Modal value:						
*STRUCTURE*	5	5	5	3	5	4
*BAPS*	11	10	15	15	9	6
*MGD*	5	5	5	3	5	5
						
Replicates *K *= 5:						
*STRUCTURE*	0.8	0.5	0.7	0.3	0.8	0.1
*BAPS*	0.0	0.0	0.0	0.0	0.0	0.0
*MGD*	0.8	0.8	0.7	0.1	1.0	0.5

See Table 2 for the explanation of scenarios

BAPS also overestimated the number of clusters in HWD and LD situations, while STRUCTURE and MGD yielded similar results in HWD situations. MGD performed better than STRUCTURE in LD situations.

When the fraction of replicates with the correct number of estimated clusters was the comparison parameter, the best performance was obtained with STRUCTURE at relative reduced levels of differentiation between subpopulations (at *m *= 0.10, in 90% of the replicates *K *= 5). Both BAPS and MGD performed poorly at low levels of *F*_*ST *_(see Table 2). However, when *K *= 10, MGD was better than BAPS and STRUCTURE. In the HIM, MGD always found five clusters but the performance of STRUCTURE was reduced. BAPS never ascertained the correct number of clusters. In the scenarios where HWD and LD were presented, BAPS never obtained the correct number of clusters. MGD performed slightly better than STRUCTURE in LD situations. However, in HWD situations, the behaviours of STRUCTURE and MGD were quite similar depending on the evaluated proportion of selfing.

**Figure 3:**
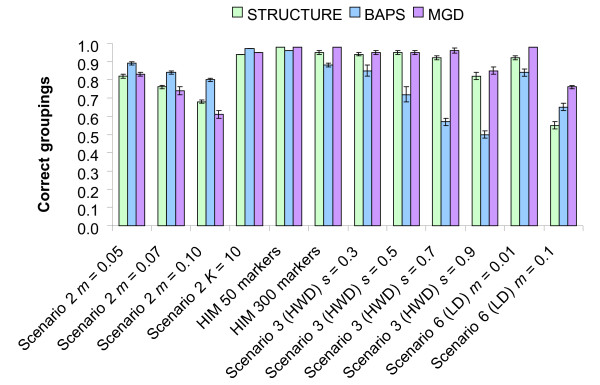
**Proportion of correct groupings with different migration rates, *K *= 10, HIM, HWD and LD**. Mean proportion of correct groupings over replicates for each simulated migration rate (*m*) and a higher number of subpopulations (*K *= 10) in scenario 2; hierarchical island model (HIM) with 50 microsatellites and 300 SNP; scenario 3 with selfing (0.3, 0.5, 0.7 and 0.9) to generate Hardy-Weinberg disequilibrium (HWD) and in scenario 6 with linked loci (recombination rate = 0.06) and 1000 generations with no migration between subpopulations and 10 generations where *m *= 0.01 or *m *= 0.1 to generate linkage disequilibrium (LD); bars represent standard errors; see Table 1 for the explanation of the scenarios.

The averaged proportion of correct groupings across the clusters with the highest membership for scenarios simulating different migration rates, *K *= 10, HIM, HWD and LD situations is shown in Figure 3. BAPS software presented a higher accuracy for all the tested differentiation levels. In the same context, no important differences were detected between STRUCTURE and MGD, though the former had a better behaviour at *m *= 0.10. The same relative performance was observed for scenario 2 and *K *= 10. In HIM, no significant differences were detected between STRUCTURE and MGD, while with BAPS a reduced proportion of correct groupings was obtained. In HWD situations no significant differences were detected between STRUCTURE and MGD, although the latter performed better. On the contrary, again with BAPS a reduced proportion of correct groupings was obtained. In LD situations, MGD performed better than STRUCTURE and BAPS.

### Randomisation procedure

In three replicates of the modified scenario 3 with *s *= 0.7 (simulated to generate HWD) and in two replicates of the modified scenario 6 with *m *= 0.01 (simulated to generate LD), STRUCTURE failed to estimate the correct number of clusters, as shown in Table 4 (*F*_*IS *_= 0.36 ± 0.10 and the mean proportion of significant loci pairs with significant linkage was 0.77 ± 0.05). Thus, these five replicates were selected as an example for the randomisation procedure to re-establish HWE and/or LE. It should be noted that BAPS failed to infer the real number of clusters in all the replicates. Then, in these five replicates, both Bayesian methods were unsuccessful. For those cases, MGD inferred five clusters except for one replicate (three clusters were determined instead) and that pattern did not change due to the randomisation.

In general, when alleles were randomised, the methods estimated the number of clusters correctly (except in one replicate with STRUCTURE) and also gave a high percentage of correct groupings (above the 98%) because HWE and LE were reached (*F*_*IS *_= - 0.01 ± 0.01 and the mean proportion of significant loci pairs with significant linkage was 0.04 ± 0.02). When only LD was present (haplotype randomisation, *F*_*IS *_= 0.00 ± 0.01 and the mean proportion of significant loci pairs with significant linkage was 0.68 ± 0.06), BAPS always overestimated the number of clusters (STRUCTURE overestimated *K *only in one replicate) and gave a mean proportion of correct groupings of 0.82 ± 0.02. When the genotypes were randomised in the modified scenario 3 (any LD removed, *F*_*IS *_= 0.36 ± 0.10 and the mean proportion of significant loci pairs with significant linkage was 0.02 ± 0.01), BAPS still overestimated the number of clusters but with a greater proportion of correct groupings of 0.87 ± 0.06. The MGD method always gave a percentage of correct groupings above 98%, whatever the randomisation option (data not shown).

### Real data

**Figure 4:**
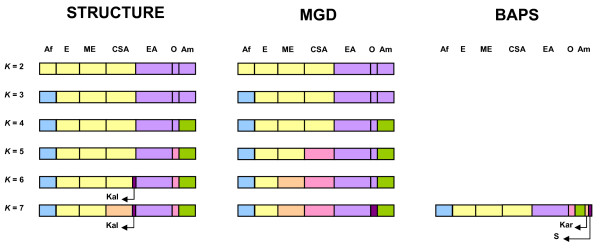
**Schematic representation of the population structure and the relationship with geographic regions in humans**. STRUCTURE results taken from Rosenberg *et al. *[34] and BAPS results from Corander *et al. *[21]; MGD: maximisation of the genetic distance method, *K*: number of inferred clusters, *N*: population size; each box corresponds to a geographical region and the width of the boxes indicates graphically the number of genotyped individuals; Af: Africa (*N *= 119), E: Europe (*N *= 161), ME: Middle East (*N *= 178), CSA: Central-South Asia (*N *= 210), EA: East Asia (*N *= 241), O: Oceania (*N *= 39), Am: America (*N *= 108), Kal: Kalash (*N *= 25), Kar: Karitiana (*N *= 24), S: Surui (*N *= 21); black lines separate regional affiliations (on the top of the figure) of the individuals; for each analysed *K *the partition obtained with each methodology is represented with *K *different colours.

A schematic representation of the correspondence between the inferred population structure and the geographic regions in the real data set using STRUCTURE [34], BAPS [21] and MGD is shown in Figure 4. The results provided by STRUCTURE suggest that the optimal structure comprised five groups that seemed to correspond well to five major geographic regions excluding an outlier, the Kalash population. When *K *= 7, STRUCTURE separated Central-South Asia. BAPS results coincided closely with the results obtained with STRUCTURE; however, it suggests a separation in more groups, allocating the populations from America in three divergent groups. The MGD partition was, in general, equal to STRUCTURE for *K *= 2 to *K *= 4, with this value being optimal under the new method. When *K *= 5, STRUCTURE distinguished Oceania while MGD divided Central-South Asia. If *K *= 6, MGD separated the Middle East completely. When *K *= 7, MGD suggested the seven main evaluated geographic regions (Africa, Europe, Middle East, Central-South Asia, East Asia, Oceania and America).

## Discussion

Clustering approaches allow the partition of a sample of individuals into genetically distinct groups without an *a priori *definition of these groups. Most of the recent advances in clustering methodology have been made using Bayesian statistical models [3,20,5,22]. Bayesian methods assign individuals to groups based on their genotypes and the assumption that the markers are in Hardy-Weinberg and linkage equilibrium within each subpopulation.

In this study, a new method was used to infer the hidden structure in a population, based on the maximisation of the genetic distance and not making any assumption on HWE and LE, and we show that it yields a good performance under different simulated scenarios and with a real data set. Therefore, it could be a useful tool to determine genetically homogeneous groups, especially in those situations where the number of clusters is high, with complex population structure and where HWD and/or LD are present.

The simulation results indicate that the BAPS method is the least precise since it needed a large number of genotyped markers to reach the correct partition, especially when the population had reached the mutation-migration-drift equilibrium. For the original/basic scenarios, the performances of MGD and STRUCTURE were similar (good) whatever the parameter of comparison, although the new method presented a slight advantage (see Table 3 and Figure 2).

We have shown that departures from the implicit assumptions in the Bayesian methods about the Hardy-Weinberg and linkage equilibrium within populations affect their accuracy, especially for BAPS, leading to an overestimated number of clusters and a reduced proportion of correct groupings. These observations are in agreement with Kaeuffer *et al. *[35] who have shown that a high LD correlation coefficient value increases the probability of detecting spurious clustering with STRUCTURE. The randomisation of alleles (and also the randomisation of genotypes and haplotypes to some extent) re-establishes both HWE and LE. In these situations, the two methods evaluate correctly the number of clusters and give an increased proportion of correct groupings. On the contrary, MGD is more precise in disequilibrium situations and its performance does not change significantly after the randomisation, demonstrating the independence of the novel method from the existence or not of HWE and LE. From the results presented here, an alternative to test the accuracy of the results from any clustering method would be to compare the results obtained after the randomisation of the molecular information within each pre-defined subpopulation when this information is available.

The precision of all three methods is excellent for *F*_*ST *_as low as 0.03. This is in agreement with the results of Latch *et al. *[10], who have proven that STRUCTURE and BAPS discern the population substructure extremely well at *F*_*ST *_= 0.02 - 0.03. However, in our simulations only STRUCTURE determines the correct number of clusters at *F*_*ST *_= 0.01. Notwithstanding, there is a controversy about the minimum differentiation level necessary for a population to be considered as genetically structured. Waples and Gaggiotti [36] have suggested that if *F*_*ST *_is too reduced (*e.g. F*_*ST *_= 0.01) then it probably cannot be associated with statistically significant evidence for departures from panmixia. In these situations, it is not clear if the most appropriate solution for MGD (and also the other clustering methodologies) is to separate different subpopulations or to maintain the subpopulations as an undifferentiated population.

The simulated scenarios taking into account different selfing rates indicated both an increase in differentiation between subpopulations (*i.e. *higher *F*_*ST *_values) and an increase in Hardy-Weinberg disequilibrium (*F*_*IS *_moves from 0.01 to 0.81). However, the increase in *F*_*ST *_values (from 0.27 to 0.42) are are not as great as that of the *F*_*IS *_values indicating that the Hardy-Weinberg disequilibrium can not be masked by the effect of the differentiation level. In addition, the increase in *F*_*ST *_values should help to distinguish the different clusters and, therefore, the HWD should reach at least the lowest limit of its effect.

Our results obtained with the MGD method from the human data set are, in general, similar to those obtained with STRUCTURE [34] and also in concordance with a more recent study of 525910 SNP [37], although some discrepancies exist with the results of Li *et al. *[38] using 650000 SNP. Rosemberg *et al*. [34] have indicated multiple clustering solutions for *K *= 7 with STRUCTURE. However, the results obtained with MGD for *K *= 7 are in complete agreement with the seven geographical regions. A careful inspection of the results detects clusters where grouped individuals have multiple sources of ancestry, especially those in the Middle East and Central-South Asia. This situation (*i.e. *the estimated mixed ancestry) could be due either to recent admixture or to shared ancestry before the divergence of two populations but without subsequent gene flow between them. It has been indicated that global human genetic variation is greatly influenced by geography [39-41]. In addition, Serre and Pääbo [42] have indicated that the clusters obtained by Rosenberg *et al*. [34] have been generated by heterogeneous sampling and that these would disappear if more populations were analysed.

In this study, a simple island model with constant population sizes and invariant symmetrical migration has been considered, which are unlikely in natural systems. The performance of STRUCTURE has been recently evaluated [23] by simulating various dispersal scenarios and it seems to perform well with more complex population structures than the finite island model (hierarchical island model, contact zone model). In this study, the performance of the MGD method was better than that of the Bayesian approaches in the simulated scenarios with a higher number of clusters and a more complex population structure. However, further investigations are required to determine the capacity of the MGD method to deal with other kinds of population structure.

Computation time may be a limitation of the new method, especially when dealing with large amounts of markers. However, it should be noted that clustering analysis is not performed very often and the results are not usually needed urgently. Therefore, it may be worthwhile to wait for the results obtained with the most accurate method.

If the genetic distance calculated from the molecular coancestry has been evaluated as an alternative, then the use of other genetic distances previously published in the literature [24] could be investigated as the parameter to maximise both for codominant and dominant molecular markers. Moreover, the Nei minimum distance [25] could be inappropriate when working with various markers, for example when mixing data obtained with markers with different heterozygosis levels (*e.g. *mixing microsatellite and SNP data). In addition, a weighting procedure [43,44] could also be implemented taking into account the subpopulation size, the number of loci or the number of alleles. Notwithstanding, the nature of the new method (*i.e. *the maximisation of the genetic distance) allows for the use of any measure which could better fit the available molecular data, beyond the Nei distance.

The informativity of the markers has a clear effect on the efficiency of the clustering methods, especially for BAPS. Increasing the number of markers (scenario 1 vs. 2, 3 vs. 4, 5 vs. 6 and 7 vs. 8) almost always yields better results: the correct number of clusters is estimated in more cases and the percentage of correct groupings is higher. In parallel, when comparing a similar number of markers but with different degrees of polymorphism (scenario 2 vs. 5, microsatellites vs. SNP) the biallelic markers yield worse performances. Notwithstanding, when using a reasonable number of markers (50 microsatelites and 300 SNP) MGD and STRUCTURE, at least, provide a high accuracy. However, when comparing results obtained with STRUCTURE, it is surprising that this method showed less accuracy with 10 microsatellites than with 50 microsatellites.

Although in the present work the method has been developed for co-dominant markers, whatever the approach (molecular coancestry or allelic frequencies), the methodology can also be easily extended to dominant molecular markers by replacing the molecular coancestry matrix with a matrix of any available measure of similarity for dominant markers [45] or estimating the allelic frequencies from recessives (see [46] and references therein) and then using the typical genetic distances.

The present formulation of the method does not explicitly account for the presence of admixed individuals. To do so, a different set of probabilities should be given to each locus in each individual (in the allelic frequencies approach) allowing for each locus to be assigned to different clusters. The increase in computation time and the ability of the optimisation algorithm to deal with a larger space of solutions deserve further investigations.

A compiled file of the code used to infer the number of clusters and the assignment of the individuals to each cluster in a given sample from the molecular coancestry matrix or the allele frequencies will be available on the web site http://www.uvigo.es/webs/c03/webc03/XENETICA/XB2/Jesus/Fernandez.htm.

## Conclusion

In this study, a new method to infer the hidden structure in a population, based on the maximisation of the genetic distance and without making any assumption on HWE and LE, performed well under different simulated scenarios and with a real data set. Therefore, this could be a useful tool to determine genetically homogeneous groups, especially in those situations where the number of clusters is high, with complex population structure and where HWD and/or LD are present.

## Competing interests

The authors declare that they have no competing interests.

## Authors' contributions

STRR, MAT, and JF carried out the analysis and drafted the manuscript. All authors read and approved the final manuscript.
